# Community-based intervention to improve dietary habits and promote physical activity among older adults: a cluster randomized trial

**DOI:** 10.1186/1471-2318-13-8

**Published:** 2013-01-23

**Authors:** Mika Kimura, Ai Moriyasu, Shu Kumagai, Taketo Furuna, Shigeko Akita, Shuichi Kimura, Takao Suzuki

**Affiliations:** 1 Center for Health Promotion, International Life Sciences Institute Japan, Nishikawa Bldg., 3-5-19 Kojimachi, Chiyoda-ku, Tokyo, 102-0083, Japan; 2 Faculty of Health Sciences, University of Human Arts and Sciences, 1288 Magome, Iwatsuki-ku, Saitama City, Saitama, 339-8539, Japan; 3 Department of Physical Therapy, School of Health Sciences, Sapporo Medical University, S1 W17, Chuo-ku, Sapporo, 060-8556, Japan; 4 National Center for Geriatrics and Gerontology, 35 Gango, Morioka-machi, Obu City, Aichi, 474-8511, Japan

**Keywords:** Social health program, Community-dwelling older adults, Dietary variety, Physical activity, Self-rated health

## Abstract

**Background:**

The fastest growing age group globally is older adults, and preventing the need for long-term nursing care in this group is important for social and financial reasons. A population approach to diet and physical activity through the use of social services can play an important role in prevention. This study examined the effectiveness of a social health program for community-dwelling older adults aimed at introducing and promoting physical activity in the home at each individual’s pace, helping participants maintain good dietary habits by keeping self-check sheets, and determining whether long-standing unhealthy or less-than-ideal physical and dietary habits can be changed.

**Method:**

This cluster randomized trial conducted at 6 community centers in an urban community involved 92 community-dwelling older adults aged 65–90 years. The intervention group (3 community centers; n = 57) participated in the social health program “Sumida TAKE10!” which is an educational program incorporating the “TAKE10!® for Older Adults” program, once every 2 weeks for 3 months. The control group (3 community centers; n=35) was subsequently provided with the same program as a crossover intervention group. The main outcome measures were changes in food intake frequency, food frequency score (FFS), dietary variety score (DVS), and frequency of walking and exercise. The secondary outcome measures were changes in self-rated health, appetite, and the Tokyo Metropolitan Institute of Gerontology (TMIG) Index of Competence score.

**Results:**

Compared to baseline, post-intervention food intake frequency for 6 of 10 food groups (meat, fish/shellfish, eggs, potatoes, fruits, and seaweed), FFS, and DVS were significantly increased in the intervention group, and interaction effects of FFS and DVS were seen between the two groups. No significant differences were observed between baseline and post-intervention in the control group. Frequency of walking and exercise remained unchanged in both groups, and no significant difference in improvement rate was seen between the groups. Self-rated health was significantly increased in the intervention group. Appetite and TMIG Index of Competence score were unchanged in both groups.

**Conclusions:**

The social health program resulted in improved dietary habits, as measured by food intake frequency, FFS, and DVS, and may improve self-rated health among community-dwelling older adults.

**Trial registration number:**

UMIN000007357

## Background

The fastest growing age group globally is older adults, and in Japan the percentage of older adults has increased fourfold since 1960, from 5.7% in 1960 to 23.3% in 2011 [1]. Health promotion for older adults and the prevention of long-term care are important factors in maintaining a sound society. A population approach to diet and physical activity through the use of social services can play an important role in prevention, reach a large portion of the population, and be cost effective [2]. The health benefits of physical activity have been scientifically confirmed in older adults [3,4], and many community-based interventions on physical activity have shown improved physical function [5,6], improved cognitive function [7], reduced risk of falling [8,9], reduced decline in health-reported quality of life [10], and reduced healthcare costs[11]. Based on the scientific evidence available, the World Health Organization (WHO) [12], the American College of Sports Medicine, the American Heart Association [13], and other health related organizations have published specific recommendations on physical activity for older adults. However, the number of community-based interventions on diet has been relatively small, and nutritional intervention commonly consists of individual nutritional checkups and individual dietary counselling, or nutritional education from professionals on specific nutrients and supplements [14-17]. However, in a super-aging society like Japan, too few resources are available to provide professional advice for every individual and therefore simple health programs that promote a healthy diet are needed for community-dwelling older adults.

Against this background, we developed the “TAKE10!® for Older Adults” program for community-dwelling older adults to introduce and promote physical activity in the home at each individual’s pace and to help participants maintain good dietary habits by keeping self-check sheets, even in the absence of professional advice [18]. The purpose of this study was to examine the effectiveness of a social program held at community centers that used the TAKE10!® for Older Adults program, and determine whether it could change long-standing unhealthy or less-than-ideal physical and dietary habits. We conducted a cluster randomized trial to avoid contamination across individuals and to eliminate any access barriers to participation in this intervention program [19-21].

### TAKE10!® for Older Adults

“TAKE10” stands for eating regularly from 10 food groups and taking 10 min of physical activity at least 2–3 times per day. The program was developed in order to help community-dwelling older adults introduce physical activity into their lives and encourage their intake of a variety of foods. Physical capability, dietary habits, health status, and living environment among older adults vary greatly among individuals, and the program was designed to help older adults adjust the strength and frequency of their exercise as well as their food intake to correspond to individual capabilities and preferences. The contents of the TAKE10!® booklet are shown in Additional file 1: Appendix 1.

The program for physical activity recommends walking, stretching, muscle strengthening, and balance training in the home environment at the individual’s own pace. There are 10 simple stretching exercises in total (e.g., upward, side, hamstring, hip, Achilles tendon, and quadriceps stretching) as well as 8 muscle strengthening exercises (e.g., plantar flexions, knee flexions, side leg raises, squats, and sit-ups). None of the exercises require equipment and therefore can easily be performed at home. WHO recommends at least 150 minutes of moderate-intensity aerobic physical activity throughout the week. TAKE10!® sets the initial goal slightly lower for older adults who have no established physical activity habits. We prepared a “TAKE10!® Calendar” record to enable participants to engage in physical activity by themselves (Additional file 2: Appendix 2).

The dietary program focuses on dietary variety. Some studies have shown that dietary variety is associated with health status in older adults and therefore can be used to indicate nutritional status [22-24]. One of the simple ways to promote a balanced intake of nutrients is having dietary variety, and such variety may be a good indicator of healthy dietary habits. Over consumption of energy-dense foods, which are nutrient-poor and high in fat, sugar, and salt, and inadequate consumption of fruits and vegetables are risk factors associated with an increased incidence of non-communicable diseases [2]. Moreover, inadequate protein intake causes adverse changes in the morphology and function of skeletal muscle in older adults [25,26]. We define a healthy diet for older adults as a well-balanced diet, and promote their intake of a variety of foods using a table of 10 food groups that correspond to the 10 main food groups of the Japanese diet without rice, namely meat, fish and shellfish, eggs, milk, soybean products, green and yellow vegetables, potatoes, fruits, seaweed, and fats and oils. For this purpose, we developed the “TAKE10!® Check Sheet”(Additional file 3: Appendix 3) to allow older adults to check the variety of foods in their diet quickly and easily, and ultimately improve their dietary habits, with an overall goal of maintaining good dietary habits.

In addition, information for older adults on subjects such as oral care, incontinence, and food safety are included in the TAKE10!® booklet (Additional file 1: Appendix 1).

## Methods

### Participants

The study was conducted at 6 community centers, each with >90 m^2^ of floor space and air-conditioning, in Sumida Ward in the Tokyo metropolitan area. Inclusion criteria were (1) participation in the “Sumida TAKE10” social health program conducted by Sumida Ward, (2) age ≥65 years; (3) understanding of the main study objectives and provision of informed consent, and (4) ability to travel independently to the closest participating community center. Exclusion criteria were (1) heart attack or stroke within the previous 6 months; (2) acute hepatic dysfunction or chronic active viral hepatitis, (3) fasting blood glucose >200 mg/dl, (4) diastolic blood pressure >180 mmHg and/or systolic blood pressure >100 mmHg, and (5) medical advice prohibiting exercise. Candidates were recruited by Sumida Ward through notifications printed in the Sumida Ward Bulletin delivered to all homes. A questionnaire on demographic, dietary, physical, and lifestyle characteristics was administered to all participants by researchers and program staff before and after intervention at each community center. Body weight and height were measured at the community centers by researchers and staff.

### Sample size

Because a clinically meaningful difference in our main outcome measures could not be determined, only a provisional sample size was used. We estimated that individual randomization would require 36 participants per group for a trial with 80% power to detect a 10% difference between groups, with a 5% significance level. We assumed an intracluster correlation of 0.02 and 20 participants for each community center. Under these assumptions, we increased the sample size to 50 per group (design effect, 1.38) and cluster size was determined to be 3 per group.

### Randomization

Randomization was conducted at the community center level to avoid contamination and to eliminate access barriers to participation in this program [19-21]. The 6 community centers in Sumida Ward (total area 13.75 km2) were randomized into the intervention group (3 centers) and control group (3 centers) using opaque envelopes by a public officer who was not involved in this study. Environmental factors such as access to transportation, public services, facilities, and basic culture around each of the 6 community centers did not differ substantially and all 6 centers are within a 2-kilometer radius of each other.

Effective blinding was not possible because both the subjects and researchers clearly understood the differences between the two groups.

### Participant flow

**Figure 1:**
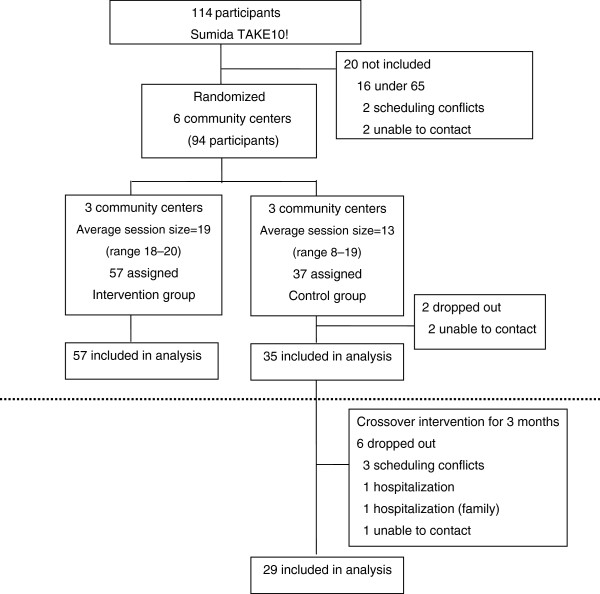
Participant flow.

The flow of participants is shown in Figure 1. From the 141 candidates for this study who were community-dwelling older adults living in Sumida Ward and participating in Sumida TAKE10!, 20 did not satisfy the following inclusion criteria: aged <65 years (n = 16), scheduling conflicts (n = 2), and unable to contact (n = 2). The remaining 94 participants were assigned to the intervention group (3 community centers; n = 57) or control group (3 community centers; n = 37) according to their home addresses. Baseline data was collected in October 2005 post-intervention data in January 2006. Two participants dropped out of the control group and 2 could not be contacted. Eventually, complete data were obtained for 57 participants in the intervention group and 35 participants in the control group.

### Intervention

Six community centers were randomized to 3 community centers for the intervention group and 3 community centers for the control group. Participants in the intervention group participated in the Sumida TAKE10! program held from October 2005 to December 2006. Participants in the control group were required only to answer the questionnaire at the same time as the intervention group, and following the intervention, the control group was provided with the same Sumida TAKE10! program as a crossover intervention group to avoid any disadvantage and also to confirm whether the effect of the program could be verified.

The Sumida TAKE10! educational social health program was aimed at helping to prevent or delay the need for long-term nursing care. It consisted of a general lecture by a researcher on the importance of dietary variety and 5 educational sessions (1.5 hours each, held once every 2 weeks) lead by researchers and staff and held at each community center. The same researchers and staff conducted the same intervention program at each community center. The sessions, which were based on the TAKE10!® program, were comprised of approximately a 30-minute lecture on practicing good dietary habits followed by 1 hour of exercise. At the first session, each participant received an explanation on how to use the TAKE10!® Check Sheet and were then required to check their diet for the following 10 days so they could gain a better understanding of their dietary habits. At the second session, participants brought the check sheets with them and analyzed the sheets themselves in the lecture to determine which food groups were not well represented and they were encouraged to increase their intake from these food groups. The check sheet was submitted every session and returned the following session with simple comments such as “Good!” “Better than before!” and “Keep up the good work!”

During the exercise program, participants were instructed in the proper way to perform each stretching and muscle strengthening exercise and the reasoning behind each exercise. After instruction, each stretching exercise was performed 2 times to the right and to the left, and muscle strengthening exercises were each performed 3–5 times at a slow pace. Researchers and staff assisted all participants who did not currently have any physical activity habits. Exercise at home by walking at a self-determined pace, stretching daily, and doing muscle strength training once every two days was recommended. Participants recorded their daily TAKE10! exercise, or lack of exercise, on the TAKE10!® Calendar and submitted it once a month. After review, we returned the Calendar with basic comments as for the TAKE10!® Check Sheet. In all sessions, priority was placed on following any instructions from the participant’s primary physician.

In this study, we aimed to determine whether decades-long habits of community-dwelling older adults could be changed by means of the TAKE10!® for Older Adults program, without receiving detailed individualized professional advice.

### Main outcomes: dietary and physical activity habit outcomes

We evaluated changes in food intake (frequency of intake of the 10 food groups) and physical activity (frequency of walking and exercise). Food intake was assessed using a questionnaire on food intake frequency covering 1 week and covering the main 10 food groups in the Japanese diet mentioned above. There were 4 choices for food intake frequency for each food group: 1) eat almost every day (3 points), 2) eat 3 or 4 days a week (2 points), 3) eat 1 or 2 days a week (1 point), and 4) eat hardly ever (0 point). We then calculated a food frequency score (FFS) as the sum of scores for each of the 10 food groups to evaluate dietary habits (range 0–30). Dietary variety score (DVS) was also calculated to evaluate dietary habits [27] and was the sum of the number of times each respondent answered “eat almost every day” for the 10 food groups (maximum score 10).

In a previous study, a higher DVS was associated with a reduced risk of higher-level functional decline [27], as determined using the Tokyo Metropolitan Institute of Gerontology (TMIG) Index of Competence score [28]: relative to a reference group with a DVS of 1–3, groups with a DVS of 4–8 or 9–10 showed significantly lower declines TMIG Index of Competence scores over a period of 5 years [27].

Frequency of physical activity was assessed using the questionnaire to determine the frequency of walking and of stretching and muscle strengthening exercise over a 1-week period.

### Secondary outcomes: health and health practice outcomes

Data on self-rated health, appetite, and higher-level functional capacity were obtained with the questionnaire. Higher-level functional capacity was measured using the TMIG Index of Competence, a multidimensional 13-item index comprising 3 subscales of instrumental self-maintenance (IADL, 5 items), intellectual activity (4 items), and social roles (4 items). Each item was scored 1 for ‘yes ‘(able to do) and 0 for ‘no’. The TMIG Index of Competence has been verified for validity and reliability, and it is widely accepted in Japan [28].

### Data analysis

Data were analyzed on an intention-to-treat basis. Mean and standard deviation (SD) was calculated for each variable. We compared the baseline characteristics of the intervention and control groups (between-group) using Student’s t-test for continuous variables, a Chi-square test, and Fisher’s exact test for proportional variables and Mann-Whitney’s U-test for categorical variables. Baseline and post-intervention data were compared as follows: FFS, DVS, and the TMIG Index of Competence within each group using a paired t-test; frequency of intake of each food group, walking and exercise frequency, and self-rated health within each group using the Wilcoxon signed-rank test; and appetite within each group using McNemer’s test. Fisher’s exact test was used to compare the positive change in the DVS subgroup scoring 1–3 between the two groups. We compared differences in the improvement rate of walking and exercise frequency, and self-rated health between the groups using the Z-test. Interaction effects were analyzed using a two-way repeated measure analysis of variance. All data were analyzed using SPSS version 11.5 J for Windows XP, and the level of significance was set at 5%.

The study was approved by the Ethics Committee of Showa Women’s University, Tokyo, Japan. All subjects provided written informed consent before being enrolled in the study.

## Results

### Baseline characteristics and attendance rate

Table 1 compares the baseline characteristics between the intervention and control groups. Compared with the control group, the intervention group had a significantly higher score (p = 0.014) for social roles on the TMIG Index of Competence, but no other significant differences were seen between the two groups. The participants were predominantly female (79.8%) as is typical for social programs in Japan [29], and all participants were previously unaware of the TAKE10! program. The mean attendance rate for the intervention classes was 68.1% (range 41– 95%). Eight subjects attended only the first general lecture on importance of dietary variety because of a lack of interest in the exercise programs (n = 3) and schedule conflicts (n = 5). Forty-one subjects (71.9%) participated in more than 3 sessions.

**Table 1:** Demographic characteristics and functional capacities of participants

**Characteristics**	**Intervention (n = 57)**	**Control (n = 35)**	***P***
**Sex, n (%)**			
**Men**	9 (15.8)	8 (22.9)	0.419^a^
**Women**	48 (84.2)	27 (77.1)	
**Age in years, mean** ± **SD**	74.3 ± 5.9	74.3 ± 5.0	0.969^c^
**BMI, mean** ± **SD**	24.3 ± 2.7	24.3 ± 3.1	0.941^c^
**Preexisting conditions, n (%)**	20 (35.1)	11 (31.4)	0.718^a^
**Hypertension**	16 (28.1)	9 (25.7)	0.805^a^
**Diabetes mellitus**	4 (7.0)	2 (5.7)	1.000^b^
**Joint pain (arthritis)**	3 (5.3)	2 (5.7)	1.000^b^
**Heart disease**	4 (7.0)	4 (11.4)	0.474^b^
**Cerebrovascular disease**	1 (1.8)	0 (0.0)	1.000^b^
**Have experienced falls, n (%) **	7 (12.3)	5 (14.3)	0.762^b^
**Lifestyle**			
**Ability to walk 1 km (yes, n (%))**	52 (91.2)	31 (88.6)	0.727^b^
**Hobby activity (yes, n (%))**	48 (84.2)	28 (80.0)	0.605^a^
**Volunteer activity (yes, n (%))**	49 (86.0)	30 (85.7)	1.000^b^
**Older adult’s group activity (yes, n (%))**	47 (82.5)	29 (82.6)	0.961^a^
**Friendly conversation with neighbors**	45 (78.9)	27 (77.1)	0.839^a^
**(2 days or more/w, n (%))**			
**Going out (2 days or more/w, n (%))**	56 (98.2)	34 (97.1)	1.000^b^
**Appetite (yes, n (%))**	55 (96.5)	32 (91.4)	0.365^b^
**Food Frequency Score (FFS), mean** ± **SD**	21.5 ± 3.7	21.1 ± 5.3	0.690^c^
**Dietary Variety Score (DVS), mean** ± **SD**	4.2 ± 2.3	3.9 ± 2.9	0.577^c^
**TMG-Index of competence, mean** ± **SD**	12.4 ± 1.1	11.9 ± 1.4	0.062^c^
**Instrumental self-maintenance, mean** ± **SD**	4.9 ± 0.2	4.9 ± 0.3	0.538^c^
**Intellectual activity, mean** ± **SD**	3.7 ± 0.6	3.8 ± 0.5	0.792^c^
**Social roles, mean** ± **SD**	3.8 ± 0.5	3.3 ± 1.1	0.014^c^
**Self-rated health, n (%)**			0.657^d^
**Very good**	7 (12.3)	3 (8.6)	
**Good**	41 (71.9)	26 (74.3)	
**Not good**	9 (15.8)	6 (17.1)	

*SD* Standard deviations.

Chi-square test^a^ or Fisher's exact test^b^ for proportional variables.

Student’s t-test^c^ for continuous variables.

Mann-Whitney’s U test^d^ for categorical variables.

### Outcomes measures

Compared to baseline, significant increases were seen in post-intervention food intake frequency for 6 food groups (meat p = 0.002; fish/shellfish p = 0.02; eggs p = 0.01; potatoes p = 0.019; fruits p = 0.029; and seaweed p = 0.001) , FFS (p = 0.000), and DVS (p = 0.001) in the intervention group, and interaction effects of FFS (F(1, 90) = 10.582, p = 0.002) and DVS ( F(1,90) = 8.968, p = 0.004) were seen between the two groups. A significant difference was seen in the percentage of participants scoring 1–3 between the groups (p = 0.041) (Table 2), but no significant difference was observed between baseline and post-intervention in the control group (Table 2). Frequency of walking and stretching and muscle strengthening exercises did not change compared to baseline in either group and no significant differences were seen between two groups (walking, Z = 1.918; exercise, Z = 0.204) (Table 2).

**Table 2:** Main outcomes in the intervention group and the control group

**Outcomes**		**Intervention**				**Control**				**Between-groups**
**Food frequency, n (%)**		**Almost every day**	**3-4 days/week**	**0-2 days/week**	***P***	**Almost every day**	**3-4 days/week**	**0-2 days/week**	***P***	***P***
**Meat**	Pre	12 (21.1)	21 (36.8)	24 (42.1)	0.002^*a*^	9 (25.7)	13 (37.1)	13 (37.1)	1.000^*a*^	
	Post	23 (40.4)	21 (36.8)	13 (22.8)		7 (20.0)	17 (48.6)	11 (31.4)		
**Fish/Shellfish**	Pre	23 (40.4)	23 (40.4)	11 (19.3)	0.020^*a*^	17 (48.6)	11 (31.4)	7 (20.0)	1.000^*a*^	
	Post	28 (49.1)	26 (45.6)	3 (5.3)		16 (45.7)	13 (37.1)	6 (17.1)		
**Eggs**	Pre	19 (33.3)	13 (22.8)	25 (43.9)	0.010^*a*^	10 (28.6)	12 (34.3)	13 (37.1)	0.527^*a*^	
	Post	24 (42.1)	20 (35.1)	13 (22.8)		8 (22.9)	14 (40.0)	13 (37.1)		
**Milk**	Pre	29 (50.9)	10 (17.5)	18 (31.6)	0.075^*a*^	20 (57.1)	6 (17.1)	9 (25.7)	1.000^*a*^	
	Post	35 (61.4)	8 (14.0)	14 (24.6)		19 (54.3)	8 (22.9)	8 (22.9)		
**Soybean products**	Pre	30 (52.6)	17 (29.8)	10 (17.5)	0.278^*a*^	17 (48.6)	11 (31.4)	7 (20.0)	0.822^*a*^	
	Post	34 (59.6)	15 (26.3)	8 (14.0)		16 (45.7)	12 (34.3)	7 (20.0)		
**Green& Yellow vegetables**	Pre	40 (70.2)	14 (24.6)	3(5.3)	0.491^*a*^	21 (60.0)	9 (25.7)	5 (14.3)	0.782^*a*^	
	Post	42(73.7)	13 (22.8)	2(3.5)		21 (60.0)	8 (22.9)	6(17.1)		
**Potatoes**	Pre	9 (15.8)	20 (35.1)	28 (49.1)	0.019^*a*^	8 (22.9)	13 (37.1)	14 (40.0)	0.225^*a*^	
	Post	15 (26.3)	25 (43.9)	17 (29.8)		6 (17.1)	12 (34.3)	17 (48.6)		
**Fruits**	Pre	42 (73.7)	9(15.8)	6(10.5)	0.029^*a*^	16 (45.7)	9 (25.7)	10 (28.6)	0.593^*a*^	
	Post	48 (84.2)	6:(10.5)	3 (5.3)		14 (40.0)	11 (31.4)	10 (28.6)		
**Seaweeds**	Pre	15 (26.3)	23 (40.4)	19 (33.3)	0.001^*a*^	7 (20.0)	17 (48.6)	11 (31.4)	0.674^*a*^	
	Post	28 (49.1)	21 (36.8)	8 (14.0)		10 (28.6)	13 (37.1)	12 (34.3)		
**Fats & Oil**	Pre	23 (40.4)	23 (40.4)	11 (19.3)	0.057^*a*^	13 (37.1)	12 (34.3)	10 (28.6)	0.858^*a*^	
	Post	33 (57.9)	16 (28.1)	8 (14.0)		10 (28.6)	17 (48.6)	8 (22.9)		
**Food Frequency Score(FFS) mean ± SD**	Pre		21.5 ± 3.7		0.000^*b*^		21.1 ± 5.4		0.631^*b*^	0.002^c^
	Post		23.9 ± 3.9				20.8 ± 4.3			
**Dietary variety Score(DVS) mean ± SD**	Pre		4.2 ± 2.3		0.001^*b*^		3.9 ± 2.9		0.328^*b*^	0.004^c^
	Post		5.4 ± 2.6				3.6 ± 2.2			
**Positive change in DVS 1–3 Score group, n (%)**			11 (55.0)				3 (18.8)			0.041^d^
		**5-7 days**	**2-4 days**	**0-1 day**	***P***	**5-7 day**	**2-4 days**	**0-1 day**	***P***	***P***
**Walking, n (%)**	Pre	35 (61.4)	7 (12.3)	15 (26.3)	0.664^*a*^	16 (45.7)	11 (31.4)	8 (22.9)	0.348^*a*^	n.s.^e^
	Post	31 (54.4)	17 (29.8)	9 (15.8)		14 (40.0)	10 (28.6)	11 (31.4)		
**Exercise, n (%)**	Pre	23 (40.4)	19 (33.3)	15 (26.3)	0.678^*a*^	17 (48.6)	6 (17.1)	12 (34.3)	1.000^*a*^	n.s.^*e*^
	Post	20 (35.1)	28 (49.1)	9 (15.8)		14 (40.0)	12 (34.3)	9 (25.7)		

Wilcoxon signed-rank test^a^ for within-groups difference of categorical variables.

Student’s t-test^b^ for between-group difference of continuous variables.

Two-way Repeated-Measures ANOVA^c^ for the time-by-group interaction of continuous variables.

Fisher's exact test ^d^ for between-group difference proportional variables.

Z-test^e^ for between-group differences of improvement rate about categorical variables. P > 0.05; Z-score > 1.96.

Self-rated health was also significantly improved over baseline in the intervention group (p = 0.033), but no difference in the improvement rate was observed between the groups. Appetite and TMIG Index of Competence score did not change between baseline and post-intervention in either group (Table 3).

**Table 3:** Secondary outcomes in the intervention group and control group

**Outcomes**		**Intervention**			***P***	**Control**			***P***	**Between-groups**
			**mean ± SD**				**mean ± SD**			***P***
**TMIG Index of Competence**	Pre		12.4 ± 1.1		0.083^*a*^		11.9 ± 1.4		0.571^*a*^	0.810^*b*^
	Post		12.5 ± 0.8				12.0 ± 1.5			
**Self-maintenance**	Pre		4.9 ± 0.2		0.146^*a*^		4.9 ± 0.3		0.422^*a*^	
	Post		5.0 ± 0.0				4.9 ± 0.2			
**Intellectual activity**	Pre		3.7 ± 0.6		0.279^*a*^		3.8 ± 0.5		0.763^*a*^	
	Post		3.9 ± 0.4				3.8 ± 0.5			
**Social roles**	Pre		3.8 ± 0.5		0.563^*a*^		3.3 ± 1.1		0.864^*a*^	
	Post		3.7 ± 0.6				3.2 ± 1.3			
		**yes, n (%)**			***P***	**yes, n (%)**			***P***	
**Appetite**	Pre		55 (96.5)		1.000^*c*^		32 (91.4)		0.625^*c*^	
	Post		56(100.0)				34 (97.1)			
		**Very good n (%)**	**Good n (%)**	**Not good n (%)**	***P***	**Very good n (%)**	**Good n (%)**	**Not good n (%)**	***P***	***P***
**Self-rated health**	Pre	7 (12.3)	41 (71.9)	9 (15.8)	0.039^*d*^	3 (8.6)	26 (74.3)	6 (17.1)	1.000^*d*^	n.s.^*e*^
	Post	12 (21.1)	40 (70.2)	5 (8.8)		3 (8.6)	26 (74.3)	6 (17.1)		

*TMIG* Tokyo Metropolitan Institute of Gerontology*, SD* standard deviation*.*

^a^Paired t-test for within-groups difference of continuous variables.

^b^Two-way Repeated-Measures ANOVA for the time-by-group interaction of continuous variables.

^c^McNemer’s test for within-groups difference of proportional variables.

^d^Wilcoxon signed-rank test for within-groups difference of categorical variables.

^e^Z-test for between-group difference of improvement rate for categorical variables. P > 0.05; Z-score > 1.96.

As shown in Tables 4 and 5, similar effects were observed for food intake frequency, FFS, DVS, and self-rated health in the crossover intervention group compared to the original intervention group. Compared to baseline, significant increases were seen in post-intervention food intake frequency for 8 food groups (meat p = 0.005; eggs p = 0.002; milk p = 0.021; soybean products p = 0.016; green & yellow vegetables p = 0.008; potatoes p = 0.003; fruits p = 0.013; and seaweed p = 0.011), FFS (p = 0.000), and DVS (p = 0.000) in the crossover intervention group. Self-rated health significantly improved (p = 0.025), and with regard to physical activity, frequency of walking did not change but frequency of exercise significantly improved in the crossover intervention group post-intervention.

**Table 4:** Main outcomes in the crossover intervention group

**Outcomes**		**Almost every day**	**3-4 days/week**	**0-2 days/week**	***P***
**Food frequency, n (%)**					
**Meat**	Pre	6 (20.7)	15 (51.7)	8 (27.6)	0.005^*a*^
	Post	13 (44.8)	12 (41.4)	4 (13.8)	
**Fish/Shellfish**	Pre	12 (41.4)	11 (37.9)	6 (20.7)	0.197^*a*^
	Post	15 (51.7)	10 (34.5)	4 (13.8)	
**Eggs**	Pre	6 (20.7)	14 (48.3)	9 (31.0)	0.002^*a*^
	Post	16 (55.2)	10 (34.5)	3 (10.3)	
**Milk**	Pre	15 (51.7)	7 (24.1)	7 (24.1)	0.021^*a*^
	Post	22 (75.9)	2 (6.9)	5 (17.2)	
**Soybean products**	Pre	13 (44.8)	11 (37.9)	5 (17.2)	0.016^*a*^
	Post	21 (72.4)	6 (20.7)	2 (6.9)	
**Green & Yellow vegetables**	Pre	17 (58.6)	6 (20.7)	6 (20.7)	0.008^*a*^
	Post	24 (82.8)	4 (13.8)	1 (3.4)	
**Potatoes**	Pre	5 (17.2)	9 (31.0)	15 (51.7)	0.003^*a*^
	Post	14 (48.3)	8 (27.6)	7 (24.1)	
**Fruits**	Pre	11 (37.9)	9 (31.0)	9 (31.0)	0.013^*a*^
	Post	18 (62.1)	5 (17.2)	6 (20.7)	
**Seaweeds**	Pre	10 (34.5)	10 (34.5)	9 (31.0)	0.011^*a*^
	Post	16 (55.2)	10 (34.5)	3 (10.3)	
**Fats & Oil**	Pre	10 (34.5)	13 (44.8)	6 (20.7)	0.115^*a*^
	Post	15 (51.7)	10 (34.5)	4 (13.8)	
**Food Frequency Score (FFS), mean ± SD**	Pre		20.9 ± 4.5		0.000^*b*^
	Post		24.7 ± 5.1		
**Dietary variety Score (DVS), mean ± SD**	Pre		3.6 ± 2.3		0.000^*b*^
	Post		6.0 ± 3.2		
**Positive change in DVS 1–3 Score group, n (%)**			7 (53.8)		
		**5-7 days**	**2-4 days**	**0-1 day**	***P***
**Walking, n (%)**	Pre	12 (41.4)	10 (34.5)	7 (24.1)	0.090^*a*^
	Post	15 (51.7)	11 (37.9)	3 (10.3)	
**Exercise, n (%)**	Pre	13 (44.8)	10 (34.5)	6 (20.7)	0.026^*a*^
	Post	21(72.4)	4 (13.8)	4 (13.8)	

Wilcoxon signed-rank test^a^ for within-groups difference of categorical variables.

Paired t-test^b^ for within-groups difference of continuous variables.

**Table 5:** Secondary outcomes in the crossover intervention group

**Outcomes**		**mean ± SD**			***P***
**TMIG Index of Competence**	Pre		12.0 ± 1.7		0.869^*a*^
	Post		12.0 ± 1.6		
**Self-maintenance**	Pre		4.7 ± 0.2		0.326^*a*^
	Post		5.0 ± 0.0		
**Intellectual activity**	Pre		3.9 ± 0.7		0.083^*a*^
	Post		3.7 ± 0.6		
**Social roles**	Pre		3.2 ± 1.4		0.846^*a*^
	Post		3.2 ± 1.2		
		**yes, n (%)f**			***P***
**Appetite**	Pre	28(96.6)			1.000^*a*^
	Post	28(96.6)			
		**Very good, n (%)**	**Good, n (%)**	**Not good, n (%)**	***P***
**Self-rated health**	Pre	1 (3.4)	23 (79.3)	5 (17.2)	0.025^*b*^
	Post	5 (17.2)	20 (69.0)	4 (13.8)	

*TMIG* Tokyo Metropolitan Institute of Gerontology, *SD* standard deviation.

^a^Paired t-test for within-groups difference of continuous variables.

^b^Wilcoxon signed-rank test for within-groups difference of categorical variables.

## Discussion

The TAKE10!® for Older Adults program at community centers appears to have improved dietary habits among community-dwelling older adults. In addition to the food intake frequency for 6 food groups, FFS and DVS were significantly increased in the intervention group, suggesting that the participants’ dietary habits changed and that dietary variety was greater than before. Increases in the frequency of intake of high-protein foods and high-fiber foods were especially positive results and may help older Japanese adults to maintain good nutritional status. There were no changes in BMI (p = 0.561) or appetite (p = 1.000) seen in the intervention group, which indicates that it was the quality not quantity of food intake in their diets that changed. The fact that 55% of participants with a baseline DVS of 1–3 improved to a post-intervention score of ≥4 indicates their risk of a decrease in higher-level functional capacity had been lowered. In addition, the interaction effects of FFS and DVS and similar results seen in the crossover intervention group indicate the efficacy of this intervention program on dietary habits.

Physical activity and good nutritional habits are important to helping community-dwelling older adults avoid or delay the need for long-term nursing care [30]. Because of difficulties in evaluating nutritional programs for older adults, few studies on such programs have been conducted to date. However, some studies have shown associations between dietary variety and nutritional status [23,24,31], quality of life [30,32], and physical and cognitive function [33,34]. It is clear that promoting dietary variety is one of the best ways to maintain proper nutritional status among older adults. Moreover, in a super-aging society like Japan, there is an urgent need for social programs that are easy to implement and follow and that do not require individual advice and attention from professionals.

It was interesting that frequency of walking and doing stretching and muscle strengthening exercises did not change even in the intervention group. Some possible reasons are that, first, the end point of this intervention was during the coldest time of year in Japan, and many people undoubtedly preferred to stay indoors. Second, at baseline, 78% participants were already in the habit of walking or engaging in exercise 5 days per week, and in this community attending radio calisthenics (“*rajio taisou*”) broadcasts in nearby parks is very popular. Third, 8 (14%) subjects did not participate in the sessions beyond the first lecture and another 8 (14%) subjects participated in fewer than 3 sessions, so they might not have been interested in our program and thus not have mastered the exercises enough to perform them at home without assistance. However, in response to the question in the post-intervention questionnaire “Did you do TAKE10 exercises at home?” 83% participants answered “Yes”, and to “How many days did you do them a week?” 78% participants answered “every 2 days or more”. In the winter, it is possible that some participants replaced their attendance of the radio calisthenics broadcasts with TAKE10 exercise as it was more difficult to go outside. In addition, significant differences were observed in the frequency of exercise in the crossover intervention group, suggesting the possibility of intervention effects on physical activity.

Self-rated health improved in the intervention group compared to baseline, although a significant difference in improvement rate was not seen between groups. Self-rated health is a global measure of health, and many studies have shown correlations with relative risk of mortality [35-38], well-being, and functional capacity [39]. For community-dwelling older adults, self-rated health is a possible indicator of quality of life. However, the observed effect may have been the result of not only attending this program, but also simply gathering together with other members of the community.

This study has several limitations. First, the study design was not an ideal randomized control trial. In order to eliminate transportation barriers to participation in this program, participants were assigned to groups according to their home address. In addition, to secure the same floor conditions at the 6 community centers, randomization was conducted before recruitment. Therefore, the two groups differed in the number of participants at baseline. However, as shown in Table 1, there were no significant differences between the two groups in the variables measured at baseline. Also, we compared the 3 baseline measures (sex, age, and TMIG Index of Competence) between the 6 clusters and no significant differences were seen. The sample size was less than the ideal 50 participants per group, and as the participants were recruited through the ward’s bulletin, participants who enrolled might have been more motivated and health conscious. This might also explain the large percentage of female participants [29]. Other recruitment methods should be considered in future studies.

Although we did not examine behavioral stage and self-efficacy, we did find some behavior changes among the participants. In response to “Did your awareness of diet change after participating in this program?” 94% participants answered “Yes”, indicating that behavioural stage or self-efficacy might have changed, although we did not evaluate this scientifically. In addition, we used the TAKE10!® Check Sheet and the TAKE10!® Calendar only as tools to motivate participants and not to measure outcomes. The tools could be used to evaluate behavioral aspects in future studies. Also, seasonal changes in participant behavior were not considered and the intervention program did not reflect this. The program started in autumn which is a good season for outdoor exercise, walking, and eating, but ended in mid-winter.

Our main outcomes on diet do not indicate the quantity of food consumed from each food group, and we did not evaluate participants’ nutritional status using biochemical indicators. From our findings, we can estimate changes in dietary habits, but cannot indicate definite effects on health. In addition, it is necessary that good habits be maintained to observe the effects. However, we did not examine how long the behavioral changes continued following the intervention. We also did not measure how much physical fitness improved as a result of the exercise training undertaken by the intervention group. Further studies are therefore needed to confirm the effects of this program.

Ultimately, we consider this intervention program to be the first step toward introducing more healthy lifestyles to community-dwelling older adults, with its focus on improving their self-management abilities and aiming to increase the health status of the community as a whole. We believe the program can serve as an important form of social support that contributes to meeting present and future healthcare challenges. Personalized programs tailored to each individual’s abilities, behavioral stage, and environment would be a good next step.

## Conclusions

The social health program conducted at community centers incorporating the TAKE10!® for Older Adults program resulted in improved dietary habits—as measured by food intake frequency, FFS, and DVS—and may improve self-rated health among community-dwelling older adults.

## Competing interests

The authors declare that they have no competing interests.

## Authors' contributions

MK, SKu, TF, SA, SKi, and TS developed the intervention program “TAKE10!® for Older Adults”. MK, AM, SKu, and SKi contributed to the conception and design of this study. MK, SKu, and SA supervised and conducted the study. MK and AM interpreted the data and prepared the paper, and TS reviewed it for accuracy. All authors contributed to reviewing and approving the final version of the paper.

## Pre-publication history

The pre-publication history for this paper can be accessed here:

http://www.biomedcentral.com/1471-2318/13/8/prepub

## Supplementary Material

Additional file 1 **Appendix 1.** Contents of the TAKE10!® booklet. Click here for file

Additional file 2 **Appendix 2.** The TAKE10!® Calendar. Click here for file

Additional file 3 **Appendix 3.** The TAKE10!® Check Sheet. Click here for file

## References

[B1] Japan Cabinet office White paper on aging society 2012 http://www8.cao.go.jp/kourei/whitepaper/w-2011/zenbun/pdf/1s1s_1.pdf

[B2] World health organization Global strategy on diet, physical activity and health 2002 http://www.who.int/dietphysicalactivity/strategy/eb11344/strategy_english_web.pdf

[B3] Paterson DH Jones GR Rice CL Aging and physical activity: evidence to develop exercise recommendations for older adults Can J Public Health 2007 98 Suppl 2 S69 S108 18213941

[B4] Paterson DH Warburton DE Physical activity and functional limitations in older adults: a systematic review rerated to Canada’s physical activity guidelines Int J Behav Nutr Phys Act 2010 7 38 10.1186/1479-5868-7-38 20459782 PMC2882898

[B5] Fitzpatric SE Reddy S Lommel TS Fischer JG Speer EM Stephens H Park S Johnson MA Physical activity and physical function improved following a community-based intervention in older adults in Georgia senior centers J Nutr Elder 2008 27 1–2 135 154 18928194 10.1080/01639360802060223

[B6] Kolbe-Alexander TL Lambert EV Charlton KE Effectiveness of a community based low intensity exercise program for older adults J Nutr Health Aging 2006 10 1 21 29 16453054

[B7] Lautenschlager NT Cox KL Flicker L Foster JK van Bockxmeer FM Xiao J Greenop KR Almeida OP Effect of physical activity on cognitive function in older adults at risk for Alzheimer disease a randomized trial JAMA 2008 300 9 1027 1037 10.1001/jama.300.9.1027 18768414

[B8] Yates SM Dunnagan TA Evaluating the effectiveness of a home-based fall risk reduction program for rural community-dwelling older adults J Gerontol A Biol Sci Med Sci 2001 56 4 M226 M230 10.1093/gerona/56.4.M226 11283195

[B9] Suzuki T Kim H Yoshida H Ishizaki T Randomized controlled trial of exercise intervention for the prevention of falls in community-dwelling older Japanese women J Bone Miner Metab 2004 22 6 602 611 10.1007/s00774-004-0530-2 15490272

[B10] Dechamps A Diolez P Thiaudiere E Tulon A Onifade C Vuong T Helmer C Bourdel-Marchasson I Effects of exercise program to prevent decline in health-related Quality of Life in highly deconditioned institutionalized older adults Arch Intern Med 2010 170 2 162 169 10.1001/archinternmed.2009.489 20101011

[B11] Aoyagi Y Shephard RJ A Model to estimate the potential for a physical activity-induced reduction in healthcare costs for the elderly based on pedometer/ accelerometer data from the Nakanojo study Sports Med 2011 41 9 695 708 10.2165/11590530-000000000-00000 21846160

[B12] World Health Organization Global recommendations on physical activity for health age group: 65 years old and abovehttp://whqlibdoc.who.int/publications/2010/9789241599979_eng.pdf 26180873

[B13] Nelson ME Rejeski WJ Blair SN Duncan PW Judge JO King AC Macera CA Castaneda-Sceppa C Physical activity and public health in older adults recommendation from the American college of sports medicine and American heart association Circulation 2007 116 9 1094 1105 17671236 10.1161/CIRCULATIONAHA.107.185650

[B14] Rydwik E Frandin K Akner G Effects of physical training and nutritional intervention program in frail elderly people regarding habitual physical activity level and activities of daily living- a randomized controlled pilot study Arch Gerontol Geriatr 2010 51 3 283 289 10.1016/j.archger.2009.12.001 20044155

[B15] Dangour AD Albala C Allen E Grundy E Walker DG Aedo C Sanchez H Flecher O Elbourne D Uauy R Effect of nutrition supplement and physical activity program on pneumonia and walking capacity in Chilean older people: a factorial randomized trial PLoS Med 2011 8 4 e1001023 10.1371/journal.pmed.1001023 21526229 PMC3079648

[B16] Hendrix SJ Fischer JG Reddy RD Lommel TS Speer EM Stephen H Park S Johnson MA Fruit and vegetable intake and knowledge increased following a community-based intervention in older adults in Georgia senior centers J Nutr Elder 2008 27 1–2 155 178 18928195 10.1080/01639360802060249

[B17] Hien VT Khan NC le Mai B Lam NT Phuong TM Nhung BT Nhien NV Nakamori M Yamamoto S Effect of community-based nutrition education intervention on calcium intake and bone mass in postmenopausal Vietnamise women Public Health Nutr 2009 12 5 674 679 10.1017/S1368980008002632 18667112

[B18] Suzuki T TAKE10!® Booklet (revised version) 2003 Tokyo: International Life Sciences Institute Japan

[B19] Snodgrass SJ Rivett DA Perceptions of older people about falls injury prevention and physical activity Australas J Ageing 2005 24 2 114 118 10.1111/j.1741-6612.2005.00086.x

[B20] Roe B Howell F Riniotis K Beech R Crome P Ong BN Older people and falls: health status, quality of life, lifestyle, care networks prevention and views on service use following a recent fall J Clin Nurs 2009 18 16 2261 2272 10.1111/j.1365-2702.2008.02747.x 19583659

[B21] McMahon S Talley KM Wyman JF Older people’s perspectives on fall risk and fall prevention programs: a literature review Int J Older People Nurs 2011 6 289 298 10.1111/j.1748-3743.2011.00299.x 22078019 PMC3268078

[B22] Marshall TA Stumbo PJ Warren JJ Xie XJ Inadequate nutrient intakes are common and are associated with low diet variety in rural, community-dwelling elderly J Nutr 2001 131 8 2192 2196 11481416 10.1093/jn/131.8.2192

[B23] Bernstein MA Tucker KL Ryan ND O’Neill EF Clemnts KM Nelson ME Evans WJ Fiatarone Singh MA Higher dietary variety is associated with better nutritional status in frail elderly people J Am Diet Assoc 2002 102 8 1096 1104 12171454 10.1016/s0002-8223(02)90246-4

[B24] Oldewage-Theron WH Kruger R Food variety and dietary diversity as indicators of the dietary adequacy and health status of an elderly population in Sharpeville, South Africa J Nutr Elder 2008 27 1–2 101 133 18928193 10.1080/01639360802060140

[B25] Evans WJ Protein nutrition, exercise and aging J Am Coll Nutr 2004 23 6 suppl 601S 609S 15640513 10.1080/07315724.2004.10719430

[B26] Thalacker-Mercer AE Fleet JC Craig BA Carnell NS Campbell WW Inadequate protein intake affects skeletal transcript profiles in older humans Am J Clin Nutr 2007 85 5 1344 1352 17490972 10.1093/ajcn/85.5.1344 PMC2447912

[B27] Kumagai S Watanabe S Shibata H Amano H Fujiwara Y Shinkai S Yoshida H Suzuki T Yukawa H Yasumura S Haga H Effects of variety on declines in high-level functional capacity in elderly people living a community Jpn J Public Health 2003 50 1117 1124 14750363

[B28] Koyano W Shibata H Nakazato K Haga H Suyama Y Measurement of competence: Reliability and validity of the TMIG Index of Competence Arch Gerontol Geriatr 1991 13 2 103 116 10.1016/0167-4943(91)90053-S 15374421

[B29] Taguchi A Murashima H Ryu S Nagata S Murashima S Characterizing active acquires and communicators of health information for health promotion intervention community Jpn J Human Ecology 2011 77 4 150 160 10.3861/jshhe.77.150

[B30] Drewnowski A Evans WJ Nutrition, physical activity, and quality of life in older adults: summary J Gerontol A Biol Sci Med Sci 2001 56 Spec No 2 89 94 11730242 10.1093/gerona/56.suppl_2.89

[B31] Position of the American Dietetic Association: nutrition, aging, and the continuum of care J Am Diet Assoc 2000 100 5 580 595 10812387 10.1016/S0002-8223(00)00177-2

[B32] Kimura Y Wada T Ishine M Ishimoto Y Kasahara Y Konno A Nakatsuka M Sakamoto R Okumiya K Fujisawa M Otsuka K Matsubayashi K Food diversity is closely associated with activities of daily living, depression, and quality of life in community-dwelling elderly people J Am Geriatr Soc 2009 57 5 922 924 10.1111/j.1532-5415.2009.02235.x 19470014

[B33] Clausen T Charlton KE Gobotswanq KS Holmboe-Ottesen G Predictors of food variety and dietary diversity among older persons in Botswana Nutrition 2005 21 1 86 95 10.1016/j.nut.2004.09.012 15661482

[B34] Wengreen HJ Neilson C Munger R Corcoran C Diet quality is associated with better cognitive test performance among aging men and women J Nutr 2009 139 10 1944 1949 10.3945/jn.109.106427 19675102 PMC2744615

[B35] Yu ES Kean YM Slymen DJ Liu WT Zhang M Katzman R Self-perceived health and 5-year mortality risks among the elderly in Shanghai, China Am J Epidemiol 1998 147 9 880 890 10.1093/oxfordjournals.aje.a009542 9583719

[B36] Mackenbach JP Simon JG Looman CW Joung IM Int J Self-assessed health and mortality could psychosocial factors explain the association? Int J Epidemiol 2002 31 6 1162 1168 10.1093/ije/31.6.1162 12540717

[B37] Spiers N Jagger C Clarke M Arthur A Are gender differences in the relationship between self-assessed health and mortality enduring? Results from three birth cohorts in Melton Mowbray, United Kingdom Gerontologist 2003 43 3 406 411 10.1093/geront/43.3.406 12810905

[B38] Okamoto K Tanaka Y Subjective usefulness and 6-year mortality risks among elderly persons in Japan J Gerontol B Psychol Sci Soc Sci 2004 59 5 246 249 10.1093/geronb/59.5.P246 15358797

[B39] Benyamini Y Leventhal EA Leventhal H Elderly people’s ratings of the importance of health-related factors to their self-assessments of health Soc Sci Med 2003 56 8 1661 1667 10.1016/S0277-9536(02)00175-2 12639583

