# Supplementary material for: Community-based intervention to improve dietary habits and promote physical activity among older adults: a cluster randomized trial
Source: BMC Geriatr. 2013 Jan 23;13:8. doi: 10.1186/1471-2318-13-8 (PMC3560222; doi:10.1186/1471-2318-13-8)
Supplement: Additional file 1 — Appendix 1. Contents of the TAKE10!® booklet. [file 1471-2318-13-8-S1.pdf]

# Contents of the TAKE10!® booklet

- How to use this booklet
- Before starting the exercise
- When you go for a walk
- Eat many kinds of foods
- Let's start the TAKE10!® Exercise!
- The 1st day Learn it!
- The 2nd day Learn it!
  - Eat 10 different food groups a day
- The 3rd day Learn it!
  - Enjoy your diet with someone else
- The 4th day Learn it!
  - Dietary guidelines for long and happy life
- The 5th day Learn it!
- The 6th day Learn it!
  - Use a microwave oven
- The 7th day Learn it!
  - Don't be embarrassed about incontinence
- The 8th day Try it! On the floor
  - Chewing and swallowing to enjoy your meal
- The 9th day Try it!
  - While watching TV
  - Tongue exercise to enjoy your meal
- The 10th day Gaining more understanding
  - Let's take a walk
  - Recommendations for safe food preservation
  - Food with health claims
- TAKE10!® Check Sheet
- Examples for 10 foods groups
- TAKE10!® Calendar
- Ms. Tenko's upgraded version
