# Supplementary material for: Community-based intervention to improve dietary habits and promote physical activity among older adults: a cluster randomized trial
Source: BMC Geriatr. 2013 Jan 23;13:8. doi: 10.1186/1471-2318-13-8 (PMC3560222; doi:10.1186/1471-2318-13-8)
Supplement: Additional file 2 — Appendix 2. The TAKE10!® Calendar. [file 1471-2318-13-8-S2.pdf]

月 TAKE10!® Calendar

テイク テン

あなたの TAKE10!®（ウォーキング、毎日牛乳を飲む、その他なんでも）

- 毎日の記録をしてみましょう。
  - 運動したら マークに○印をつけましょう （例 ）
  - 食生活チェックは何点でしたか？ （例 8 点）

日	月	火	水	木	金	土
点	点	点	点	点	点	点
点	点	点	点	点	点	点
点	点	点	点	点	点	点
点	点	点	点	点	点	点
点	点	点	点	点	点	点
