# Supplementary material for: Community-based intervention to improve dietary habits and promote physical activity among older adults: a cluster randomized trial
Source: BMC Geriatr. 2013 Jan 23;13:8. doi: 10.1186/1471-2318-13-8 (PMC3560222; doi:10.1186/1471-2318-13-8)
Supplement: Additional file 3 — Appendix 3. The TAKE10!® Check Sheet. [file 1471-2318-13-8-S3.pdf]

TAKE 10!® Check Sheet

（テイク テン）

10ページを参考に10日分の食生活の点数を記入してみましょう

	肉	魚	卵	牛乳	大豆	海草	イモ	果物	油	野菜	合計
	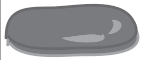	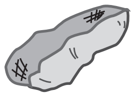	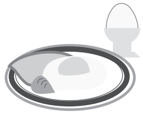	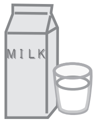	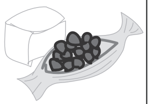	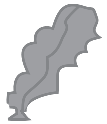	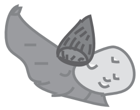	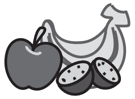	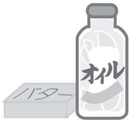	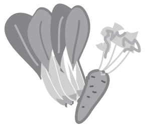	
1日目 ／											
2日目 ／											
3日目 ／											
4日目 ／											
5日目 ／											
6日目 ／											
7日目 ／											
8日目 ／											
9日目 ／											
10日目 ／											
10日間の合計											

10日分の合計点数を見てみると、あなたがどんな食品にかたよって食べているか、どんな食品をあまり食べていないかがわかります。
この点数表を参考にしてバランスのとれた食事を心がけましょう。

Copyright © 2003 ILSI Japan All Rights Reserved.
